# Energy-Based Acoustic Source Localization Methods: A Survey

**DOI:** 10.3390/s17020376

**Published:** 2017-02-15

**Authors:** Wei Meng, Wendong Xiao

**Affiliations:** 1 School of Automation and Electrical Engineering, University of Science and Technology Beijing, Beijing 100083, China; 2 Temasek Laboratories, National University of Singapore, Singapore 117411, Singapore; tslmeng@nus.edu.sg

**Keywords:** wireless sensor network (WSN), source localization, maximum likelihood method, least-squares method, Cramer–Rao bound (CRB)

## Abstract

Energy-based source localization is an important problem in wireless sensor networks (WSNs), which has been studied actively in the literature. Numerous localization algorithms, e.g., maximum likelihood estimation (MLE) and nonlinear-least-squares (NLS) methods, have been reported. In the literature, there are relevant review papers for localization in WSNs, e.g., for distance-based localization. However, not much work related to energy-based source localization is covered in the existing review papers. Energy-based methods are proposed and specially designed for a WSN due to its limited sensor capabilities. This paper aims to give a comprehensive review of these different algorithms for energy-based single and multiple source localization problems, their merits and demerits and to point out possible future research directions.

## 1. Introduction

Wireless sensor networks (WSNs) are often used for monitoring tasks, such as detection, classification, localization and tracking of one or more targets in two-dimensional (2D) or three-dimensional (3D) sensor fields [1,2,3,4,5]. Due to the limited resources for sensing, communication and computation in WSNs, energy-efficient collaborative signal processing algorithms are needed [4,6]. The localization problem has received considerable attention recently [7,8,9,10]. Source localization in wireless sensor networks (WSNs) is an important problem encountered in many indoor and outdoor applications [2,5,7,11,12,13,14].

There has been a rich history of published work that attempts to solve the source localization problem in WSNs. These solutions fall into three categories, in which the localization solutions are based on three different types of physical measurements: (1) time of arrival (TOA) [15,16,17,18,19,20] or time difference of arrival (TDOA) [21,22,23,24,25]; (2) direction of arrival (DOA) [4,26,27]; and (3) received signal strength (RSS) or energy [1,7,11,12,28,29,30,31,32,33,34,35,36,37,38].

Among three types of physical measurements, TOA and TDOA requires high precision hardware for timing purposes. Usually, in passive source localization, we do not know the propagation time. Hence, TDOA-based methods are chosen instead of TOA-based ones, however, in which the time synchronization problem arises. This needs extra calibrations. Furthermore, these time-based methods require precise acquisition of the phases of the signals arriving at different nodes. However, the features of some signals tend to be narrow-band, making precise phase acquisition difficult in a noisy environment.

DOA-based methods need sensors equipped with antenna sub-arrays, e.g., a microphone sensor in acoustic networks, each capable of independently detecting the source signal and producing a DOA bearing estimate. The crossings of these bearing estimates are then combined to produce an estimate of the most likely source location. DOA-based methods can be used for narrow-band signals. In [27], a DOA-based collaborative acoustic environmental monitoring system is developed. An embedded networked sensing box (ENSBox) platform is used, which can measure DOA from birds, etc. The method in this paper can be used to localize actual animals in their natural habitat. However, deploying many ENSBox nodes can be prohibitively costly.

Compared to the TDOA and the DOA methods, RSS- or energy-based approaches are attractive because they are widely applicable to WSNs, do not require additional hardware and can reuse the existing wireless infrastructure. Hence, in this paper, we are more interested in source localization using RSS or energy measurements in acoustic sensor networks.

For the acoustic sources, the wideband signal model may be more appropriate. Since acoustic signals are unmodulated and may contain a wider bandwidth. In many cases, we are interested in locating the source in the near-field [4]. Here, the term ”near-field” means the sensors are close to the source. In [4,26], the authors explained acoustic source localization and beamforming problems well. In their work, maximum likelihood-based methods were used to solve the DOA source localization problem using near-field wide-band acoustic signals. Recently, in [39], by using distributed asynchronous sensors, acoustic source localization was achieved using the TDOA technique. Another good work done in acoustic source localization is [40]. In this work, the authors presented a low-complexity method for acoustic event detection and localization considering a change detection statistical framework. Two possible implementation approaches based on the efficient cumulative sum (CUSUM) algorithm [41], namely CUSUM-FT (fixed time) and CUSUM-ML (maximum likelihood), were presented and discussed. For the source localization method, the TDOA technique was adopted. A real implementation was conducted to demonstrate the validity of the proposed CUSUM method in a rectangular room of dimensions of 7 m × 6 m. For noisy and reverberant environments, in [42,43,44], a wide range of acoustic source localization methods based on steered-response power (SRP) was proposed, e.g., the steered response power-phase transform (SRP-PHAT) algorithm in [43], a modified SRP-PHAT in [42], etc.

The methods for acoustic source localization presented above can be categorized as signal-based methods, which are different from energy-based ones. Energy estimates of the source are obtained at each sensor via averaging of the data samples; these single estimates are fused either in a centralized (transmitted to a fusion center) or decentralized fashion to form the final localization estimate. For some scenarios, signal-based methods may offer improved performance versus energy-based methods since the information conveyed in all samples is directly exploited (without averaging), but at the expense of larger transmission resources, e.g., wireless bandwidth [37]. The energy-based model was first presented in [7,29], which is derived from the acoustic RSS model. In essence, the energy of the signal is the average RSS measurements over the time window [t−T/2,t+T/2]. An energy-based approach for acoustic source localization is an appropriate choice since the acoustic energy emitted by the sources usually varies slowly. As such, the acoustic energy time series can be sampled at a much lower rate compared with the raw acoustic time series [7]. Therefore, few data will be transmitted to the fusion center via the often congested wireless communication channels. This will reduce the energy consumption for data transmissions at the individual sensor node and save communication bandwidth over shared wireless channels.

This paper provides a survey on the energy-based source localization in acoustic sensor networks. Most of the works in the literature focus on the single source localization case. In this paper, an overview of different methods, including weighted least-squares (WLS) [28,29,35,37], semidefinite programming [30,45], second-order cone programming (SOCP) [46] and project-onto-convex set (POCS) [38], will be provided. Furthermore, two methods for the energy-based multiple-source localization problem, i.e., the multi-resolution (MR) search method and an efficient expectation maximization (EM) method, will be introduced.

The paper is organized as follows. Section 2 briefly introduces the energy-based sensing model. Section 3 and Section 4 present the algorithms for the single and multiple source localization problems, respectively. Section 5 concludes the paper and points out possible future research directions.

## 2. Energy-Based Sensing Model

The model below is widely used for acoustic signals [7,28,29,47]. The acoustic signal received by a sensor at time instant *n* can be expressed as:

(1) $$z\left(n\right)=\gamma \frac{P(n)}{d(n)}+\epsilon \left(n\right)=s\left(n\right)+\epsilon \left(n\right),$$

where z(n) represents the acoustic intensity, *γ* is the gain factor of the sensor, P(n) denotes the intensity of the source signal measured at a location with distance of 1 m from the source, d(n) is the distance between the sensor and source and ϵ(n) is modeled as a zero-mean additive white Gaussian (AWGN) noise with variance $\varsigma^{2}$.

The energy-based model was first presented in [7,29,48], which is derived from the acoustic RSS Equation (1). In essence, the energy is the average intensity measurements over the time window [t−T/2,t+T/2].

Assume that ϵ(n) and P(n) are uncorrelated, such that:

E{P(n)ϵ(n)}=E{P(n)}E{ϵ(n)}=0.

Then, one may represent the acoustic energy as (setting $g=\gamma^{2}$, and $S\left(n\right)=E\{P^{2}\left(n\right)\}$):

(2) $$\begin{array}{c} E\left\{s^{2}\left(n\right)\right\}=\gamma^{2}\frac{E\{P^{2}\left(n\right)\}}{d^{2}\left(n\right)}=g\frac{S(n)}{d^{2}\left(n\right)}. \end{array}$$

In practice, the expectation is realized using the ensemble average over a time window $T=M/f_{s}$, where *M* is the number of sample points used for estimating the acoustic energy received by the sensor during the time interval *T* and $f_{s}$ is the sampling frequency. The average energy measurement over the time window [t−T/2,t+T/2] denoted by y(t) is given by:

(3) $$\begin{array}{c} y\left(t\right)=\frac{1}{f_{s}T}\sum_{n=\left(t-T/2\right)f_{s}}^{\left(t+T/2\right)f_{s}}z^{2}\left(n\right)=\frac{1}{f_{s}T}\sum_{n=\left(t-T/2\right)f_{s}}^{\left(t+T/2\right)f_{s}}s^{2}\left(n\right)+\frac{1}{f_{s}T}\sum_{n=\left(t-T/2\right)f_{s}}^{\left(t+T/2\right)f_{s}}\epsilon^{2}\left(n\right). \end{array}$$

Then, according to the result in Equation (2), Equation (3) can be rewritten as:

(4) $$y\left(t\right)=g\frac{S(t)}{d^{2}\left(t\right)}+w\left(t\right).$$

The square of the background noise $\epsilon^{2}\left(n\right)$ in Equation (3) will have a $\chi^{2}$ distribution with the mean equal to $E\left\{\epsilon^{2}\left(n\right)\right\}=\zeta^{2}$ and the variance equal to $2\zeta^{4}/M$. If *M* is sufficiently large (M≫30), according to the central limit theorem, *w* can be approximated well with a normal distribution, namely $w∼N(\zeta^{2},2\zeta^{4}/M)$.

## 3. Single Source Localization: Algorithms and Analysis

**Figure 1:**
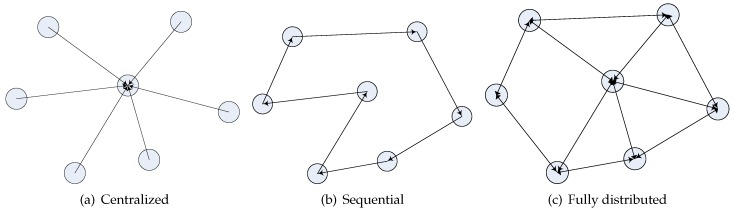
Types of source localization algorithms.

This section covers the single source localization case for energy-based methods. Considering the implementation issue, source localization algorithms can be categorized as three types: centralized, sequential and fully-distributed, as shown in Figure 1. In a centralized algorithm, as shown in Figure 1a, the sensors need to transmit the data to a central point or a fusion center for source location estimation. However, in a sequential or a fully-distributed method, no fusion center is required. As shown in Figure 1b, for the sequential algorithms, the measurements by the sensors are processed sequentially through the networks. A specified data transmitting path is needed. How to do path planning in such networks is a big issue. Furthermore, the convergence rate is low when the sensor density is high and data transmission becomes unreliable when some of the nodes fail; while in a fully-distributed algorithm, as shown in Figure 1c, at each time step, the sensors can exchange their estimates with their one-hop neighbors. All of the sensors update their estimates simultaneously, and finally, they are able to achieve consensus on a possible minimizer asymptotically. The fully-distributed algorithms avoid the path planning problem in the sequential distributed method and improve the robustness of the network.

Centralized methods: The first work on energy-based source localization was done by Li and Hu. In their papers [28,29], the energy-ratio least-squares (ER-LS) method is proposed. The first step of this method is to obtain the energy-ratio to eliminate the unknown parameter S(t). Then, in the second step, the localization of the source is solved by minimizing a nonlinear least-squares cost function. This method is an approximate solution for maximum likelihood estimation (MLE) for single source localization. Another method reported in their work is two-dimensional CPA (closest point of approach). In general, the CPA method searches for the sensor with the maximum energy reading for the single source situation. However, the estimation accuracy of this method highly depends on the density of the sensors. In [35], a two-stage algebraic closed-form solution was presented. The first stage computes the source location together with an auxiliary variable using the weighted least-squares method. The second stage explores the relationship between the source location and the auxiliary variable to improve the location estimation. In [30], a weighted direct least-squares formulation was presented in [37] that provides a tradeoff between performance and computational complexity. In [45,46,49], the source localization problem was formulated using semi-definite programming and second-order cone programming methods.

Sequential methods: In [34], Rabbat and Nowak proposed a distributed implementation of the incremental gradient (IG) algorithm to solve the nonlinear least-squares problem. However, in their work, the source energy S(t) is assumed to be known. Later in [32], they propose to use a kernel averaging estimator for robust source localization. In [38], Blatt and Hero first formulated the source localization as a convex feasibility problem (CFP) and proposed to use a projection-onto-convex sets (POCS) method. This method can be implemented in a distributed manner. However, the convergence of the method is verified by simulation, and no rigorous analysis is provided. An incremental optimization algorithm for maximum likelihood-based source localization was proposed in [36].

Fully-distributed method: In [5,13], the authors formulated the source localization problem as a convex feasibility problem (CFP) and proposed a fully-distributed method for it. In the proposed method, sensor nodes only need to communicate with one-hop neighbors and update their estimates simultaneously based on a projection algorithm. Finally, they are able to achieve consensus on a possible minimizer asymptotically. The proposed method has low complexity and can achieve global optimality fast.

In the following, we will give more details about the algorithms for the energy-based single source localization problem.

### 3.1. Centralized Algorithms

In this part, we give a brief introduction about the existing methods for centralized source localization. For the energy-based source localization using weighted least-squares method, we mainly introduce algebraic closed-form solutions proposed in [28,29,35]. The interested readers are referred to [30,37] for the alternative methods therein.

Let the source be located at an unknown coordinate pair $\theta ={\left[x,y\right]}^{T}$. We assume that there are *N* sensor nodes performing sensing based on energy detection. The location of the *i*-th sensor is denoted by $r_{i}={\left[x_{i},y_{i}\right]}^{T},i=1,2,...,N$.

Here, we reproduce the energy-based sensing model for convenience.

(5) $$y_{i}\left(t\right)=g_{i}\frac{S(t)}{d_{i}^{2}}+\varepsilon_{i}\left(t\right),$$

where $d_{i}=∥\theta -r_{i}∥$ is the Euclidean distance between the *i*-th sensor and the source. For simplicity, let us assume the mean $\zeta^{2}$ has been subtracted from Equation (5) so that $\varepsilon_{i}$ follows a zero mean Gaussian distribution with variance $\sigma_{i}^{2}=2\zeta^{4}/M$.

#### 3.1.1. Unconstrained Least-Square Method

In [29], the authors formulated the energy-based source localization as an unconstrained least-square problem. Firstly, the energy-ratio $\kappa_{ij}$ of the *i*-th and the *j*-th sensors can be computed as follows:

(6) $$\begin{array}{c} \kappa_{ij}:={\left(\frac{y_{i}/y_{j}}{g_{i}/g_{j}}\right)}^{-1/2}=\frac{∥\theta -r_{i}∥}{∥\theta -r_{j}∥} \end{array}$$

Note that for $0<\kappa_{ij}\neq 1$, all of the possible source coordinates *θ* that satisfy Equation (6) must reside on a two-dimensional hypersphere described by the equation:

(7) $$\begin{array}{c} ∥\theta -c_{ij}{∥}^{2}=\rho_{ij}^{2}, \end{array}$$

where the center $c_{ij}$ and the radius $\rho_{ij}$ of this hypersphere associated with sensor *i* and *j* are given by:

(8) $$\begin{array}{c} c_{ij}=\frac{r_{i}-\kappa_{ij}^{2}r_{j}}{1-\kappa_{ij}^{2}},\;\rho_{ij}=\frac{\kappa_{ij}∥r_{i}-r_{j}∥}{1-\kappa_{ij}^{2}} \end{array}$$

This hypersphere is called a source location hypersphere.

Consider two hyperspheres based on Equation (7):

(9) $$\begin{array}{c} ∥\theta -c_{i0}{∥}^{2}=\rho_{i0}^{2}\;\;\;{∥\theta -c_{j0}∥}^{2}=\rho_{j0}^{2}. \end{array}$$

They are formed from the sensor pairs (*i*, 0) and (*j*, 0). Subtract each side, and cancel the term ${∥\theta ∥}^{2}$; we have a hyperplane equation:

(10) $$\begin{array}{c} 2\left(c_{i0}-c_{j0}\right)\theta =\left(c_{i0}^{2}-\rho_{i0}^{2}\right)-\left(c_{j0}^{2}-\rho_{j0}^{2}\right). \end{array}$$

Substitute the definition in Equation (8); the above equation is simplified to:

(11) $$\begin{array}{c} u_{ij}\theta =\eta_{ij} \end{array}$$

which is a linear hyperplane equation with:

(12) $$\begin{array}{c} u_{ij}=\frac{2r_{i}}{1-\kappa_{i}^{2}}-\frac{2r_{j}}{1-\kappa_{j}^{2}},\;\;\;\eta_{ij}=\frac{∥r_{i}{∥}^{2}}{1-\kappa_{i}^{2}}-\frac{∥r_{j}{∥}^{2}}{1-\kappa_{j}^{2}} \end{array}$$

Then, the linear least-square cost function can be written as:

(13) $$\begin{array}{c} J_{Linear}\left(\theta \right)=\sum_{i=1}^{N-1}{∥u_{i}^{T}\theta -\eta_{i}∥}^{2} \end{array}$$

Given the coefficients, a solution of *θ* can be found in closed form since there is no constraint imposed in Equation (13) [29].

#### 3.1.2. Weighted Least-Squares Method

The unconstrained least-square mentioned above is closed-form and is computationally attractive. However, it is not able to reach the Cramer–Rao bound (CRB). In [35], a two-stage closed-form least-squares method was proposed, which can achieve CRB when the signal-to-noise ratio (SNR) tends to infinity.

From Equation (5), we can see that the measurement $y_{i}\left(t\right)$ is highly nonlinearly related to the unknown source location *θ* and source energy *S*. Generally, we need to take the energy ratio to remove the dependency of *S*. Without loss of generality, we choose Sensor 1 as the reference. Then, the ratios of the energy measurements with respect to the reference can be written as:

(14) $$\begin{array}{c} q_{i1}≃\frac{\frac{y_{1}}{g_{1}}}{\frac{y_{i}}{g_{i}}}=\frac{d_{i}^{2}}{d_{1}^{2}}\left(1+\frac{\varepsilon_{1}d_{1}^{2}}{Sg_{1}}\right)\left(1-\frac{\varepsilon_{i}d_{i}^{2}}{Sg_{i}}\right), \end{array}$$

where $\left(\varepsilon_{i}d_{i}^{2}\right)/\left(Sg_{i}\right)\ll 1$ is assumed to hold, which means the signal-to-noise ratio in the energy measurements should be large enough [35].

Expanding the right-hand side and ignoring the second-order noise term yield:

(15) $$\begin{array}{ccc} q_{i1} & = & q_{i1}^{\circ }+▵q_{i1}\\ & = & {\left(\frac{d_{i}}{d_{1}}\right)}^{2}+\left(\frac{\varepsilon_{1}d_{i}^{2}}{Sg_{1}}-\frac{\varepsilon_{i}d_{i}^{4}}{Sg_{i}d_{1}^{2}}\right), \end{array}$$

The first term is the true value of $q_{i1}$, and the second term is noise. If we collect $q_{i1},\forall i=2,...,N$ to form the vector $q=q^{\circ }+▵q={\left[q_{21},q_{31},...,q_{N1}\right]}^{T}$, then the covariance matrix of q, Q has elements:

(16) $$\begin{array}{c} Q\left[i-1,j-1\right]=\left\{\begin{array}{ccc} & & \frac{d_{i}^{2}d_{j}^{2}}{S^{2}g_{1}^{2}}\sigma_{1}^{2},\;i\neq j;\\ & & \frac{d_{i}^{4}}{S^{2}g_{1}^{2}}\sigma_{1}^{2}+\frac{d_{i}^{8}}{S^{2}g_{i}^{2}d_{1}^{4}}\sigma_{i}^{2},i=j. \end{array}\right. \end{array}$$

After obtaining the formulation in Equation (15), the source localization problem was solved by a two-stage closed-form solution proposed in [35]. The basic idea of the two-stage least-squares method is that in the first stage, *θ* and $d_{1}^{2}$ are considered as independent unknown variables. The source estimate can be obtained using the standard weighted least-squares method. Then, in the second stage, the relationship between *θ* and $d_{1}^{2}$ is explored to improve the result. The details of the algorithm can be found in [35].

In [37], the authors claimed that the errors that perturb the least-squares equations (based on energy model) are not i.i.d, zero-mean Gaussian random variables. Hence, the noise term in Equation (5) is not white, but a colored one. In [37], a weighted one-step least-squares (WOS) method and weighted direct least-squares method (WD) are proposed to solve the formulated energy-based source localization problem. The WD method yields the same the location estimate as WOS. However, WD offers lower computational complexity than WOS. Similar to the two-stage weighted least-squares method stated above, a correction technique can be used to improve the location estimation by incorporating the relationship of two dependent unknown variables, which is assumed independent in the first stage. The WD with correction (WDC) method can attain CRB for the case of a white source.

#### 3.1.3. Semidefinite Programming Method

For the centralized methods, in [45,46,49], the author formulated the energy-based source localization problem in different ways. For example, the problem was solved by using the semidefinite programming method [45], second-order cone programming relaxation (SOCP) [46].

Denoting $\Theta =\theta \theta^{T},$ and relaxing it into $\Theta ⪰\theta \theta^{T}$, then the source localization problem can be formulated using following SDP:

(17) $$\min_{\theta ,\Theta }\sum_{i=1}^{N}{\left(\frac{y_{i}}{g_{i}}f_{i}\left(\theta ,\Theta \right)-\frac{1}{N}\sum_{j=1}^{N}\frac{y_{j}}{g_{j}}f_{j}\left(\theta ,\Theta \right)\right)}^{2}$$

subject to:

(18) $$\begin{array}{c} \left[\begin{array}{cc} \Theta & \theta \\ \theta^{T} & 1 \end{array}\right]⪰0 \end{array}$$

As we can see from Equation (18), the condition for the optimization function in Equation (17) (the initial maximum-likelihood (ML) problem) is an approximated one compared to the fact that $\Theta =\theta \theta^{T}$. Hence, we can claim that the SDP is an approximated solution to ML estimation of the source localization problem. Motivated by the idea of complexity reduction while keeping the performance at an acceptable level, the nonconvex ML-based localization problem is approximated by a convex second-order cone programming (SOCP) optimization problem, which can be solved very efficiently by interior point methods [50]. The basic idea of SOCP is similar to the SDP formulation. Both SOCP and SDP can be considered as approximated ML solutions.

The SDP method perform well in terms of localization accuracy. However, its main disadvantage is that it can only be used for centralized implementations.

#### 3.1.4. Quantized Signal Energy

For practical systems, it will require the quantization of the measurements before transmission. In [51,52,53], source localization using the quantized sensor signal energy readings was considered. Different quantization strategies, e.g., equally distance-divided quantizer (EDQ) [51], were proposed for energy-aware source localization. In [52], two heuristic quantization design methods were proposed from maximum-likelihood (ML) target localization. In [53], the authors consider Byzantine attacks for the location estimation task where each sensor uses a binary quantization scheme to send binary data to the fusion center.

In these target localization methods with quantized signal energy, the location estimators adopt simple or improved grid search strategies.

### 3.2. Distributed Algorithms

In this part, firstly, we introduce the existing sequential distributed methods and fully-distributed projection methods.

Distributed algorithms include the sequential and fully-distributed methods. In the literature, the distributed algorithms are mostly formulated as a convex feasibility problem (CFP). The mathematical formulation of CFP is as follows.

Suppose that $C_{1},...,C_{N}$ are closed convex subsets in a Hilbert space with intersection *C*:

$$\begin{array}{c} C=C_{1}\cap ...\cap C_{N} \end{array}$$

Convex feasibility problem (CFP): Find some point *x* in *C*.

We call the CFP consistent if C≠∅ and otherwise call it inconsistent. The projection-based method is well studied and can be used for solving the CFP.

In this section, we firstly formulate the source problem as a CFP. Then, we will present the sequential and fully-distributed algorithms as the solutions. Note that in the distributed algorithms, the source energy *S* is assumed known a priori.

#### 3.2.1. Convex Feasibility Problem Formulation

The maximum likelihood estimator (MLE) is found by solving the nonlinear least-squares problem when the noise is Gaussian:

(19) $$\theta_{ML}^{*}=\arg\min\sum_{i=1}^{N}{\left[y_{i}-g_{i}\frac{S}{d_{i}^{2}}\right]}^{2}=\arg\min\sum_{i=1}^{N}f_{i}\left(\theta \right),$$

where $f_{i}\left(\theta \right)={\left[y_{i}-g_{i}\frac{S}{d_{i}^{2}}\right]}^{2}$. Clearly, $f_{i}\left(\theta \right)$ attains its minimum of zero on the circle:

(20) $$C_{i}=\{\theta \in R^{2}:∥\theta -r_{i}∥=\sqrt{g_{i}S/y_{i}}\}.$$

However, due to the observation noise, the source may not appear on the circles defined in Equation (20), but be included in a ring area, i.e., $\sqrt{\frac{g_{i}S}{y_{i}+\zeta \sigma_{i}}}\leq ∥\theta -r_{i}∥\leq \sqrt{\frac{g_{i}S}{y_{i}-\zeta \sigma_{i}}}$ if a ζ−σ area of the noise distribution is adopted, where *ζ* is a constant (Generally, ζ=1–3). This will lead the source localization problem being a non-convex optimization problem, which is generally difficult to handle. To overcome the difficulty, a relaxation method needs to be used. We define a convex set, which is a circular area:

(21) $$D_{i}=\left\{\theta \in R^{2}:∥\theta -r_{i}∥\leq \sqrt{\frac{g_{i}S}{y_{i}-\zeta \sigma_{i}}}\right\},$$

where only the outer ring is considered. In this paper, we set ζ=1. Intuitively, the source may or may not reside in $D_{i}$. If the source is inside $D_{i},\forall i$, then it is easy to see that the source localization problem can be solved by letting the estimator be a point in the intersection of the sets $D_{i},i=1,2,...,N$. That is,

(22) $$\hat{\theta }\in D=\overset{N}{\underset{i=1}{⋂}}D_{i}\subset R^{2}.$$

**Figure 2:**
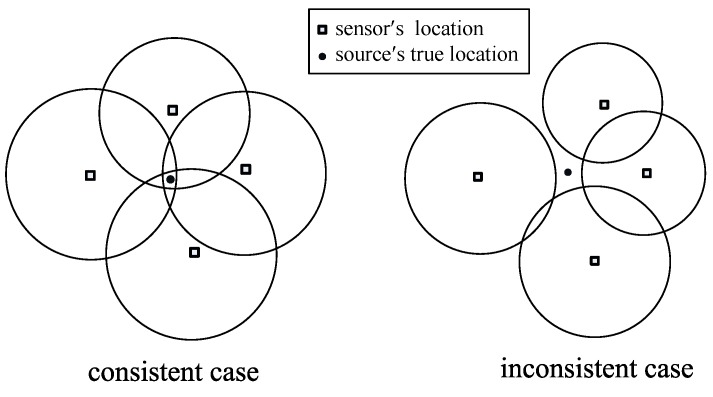
Consistent case and inconsistent case [5].

However, due to the observation noise, the feasibility problems may turn out to be inconsistent, i.e., the intersection D may be empty. An illustration for the consistent and inconsistent cases is given in Figure 2.

Since the convex feasibility problem may turn out to be inconsistent, the localization problem can be reformulated as finding a point $\theta^{*}$ that minimizes the sum of the squares of the distances to the convex set $D_{i}$.

(23) $$\begin{array}{c} \theta^{*}=\arg\min_{\theta \in R^{2}}\sum_{i=1}^{N}{∥\theta -P_{D_{i}}\left(\theta \right)∥}^{2}, \end{array}$$

where for a closed convex set *S* $\subseteq R^{2}$ and vector $x\in R^{2},P_{S}\left(x\right)$ is the orthogonal projection of *x* onto *S*. That is,

(24) $$P_{S}\left(x\right)=\arg\min_{y\in R^{2}}∥x-y∥,y\in S.$$

For the source localization problem, the projection operator in Equation (24) has a closed-form expression given as:

(25) $$P_{D_{i}}\left(x\right)=\left\{\begin{array}{ccc} & & x,\;∥x-r_{i}∥\leq \sqrt{\frac{g_{i}S}{y_{i}-\zeta \sigma_{i}}};\\ & & r_{i}+\sqrt{\frac{g_{i}S}{y_{i}-\zeta \sigma_{i}}}\frac{x-r_{i}}{∥x-r_{i}∥},\mathrm{otherwise}. \end{array}\right.$$

It can be easily checked that if the problem is consistent, i.e., $D={⋂}_{i=1}^{N}D_{i}\neq \emptyset$, then $\sum_{i=1}^{N}∥\hat{\theta }-P_{D_{i}}\left(\hat{\theta }\right)∥=0$, where $\hat{\theta }\in G$ and $G={⋂}_{i=1}^{N}D_{i}$.

#### 3.2.2. Sequential Algorithm

The sequential projection method, also termed POCS (projection on convex sets), is a cyclic algorithm [38]. From Figure 1b, we can see that the data are processed across sensors in sequence. The method is used for the energy-based source localization problem in [38]. The update rule of the sequential projection method is given as follows:

(26) $$\begin{array}{c} \theta \left(k+1\right)=\theta \left(k\right)+\lambda \left(k\right)\left[P_{D_{\tau (k)}}\left(\theta \left(k\right)\right)-\theta \left(k\right)\right] \end{array}$$

where {λ(k)} is a sequence of relaxation parameters satisfying for all $k,\epsilon_{1}\leq \lambda \left(k\right)\leq 2-\epsilon_{2}$ for some $\epsilon_{1},\epsilon_{2}>0,\tau \left(k\right)=k$ mod *N*. The pseudocode of the sequential projection method is given by Algorithm 1.

**Theorem** **1.** If $D={⋂}_{i=1}^{N}D_{i}\neq \emptyset$, any sequence θ(k),k≥0 converges to a point in D [54].

**Algorithm 1:** Sequential projection method
(1)Initialization: Set θ(0) to be an arbitrary value.(2)Iteration step: k≥0
$$\begin{array}{c} \theta \left(k+1\right)=\theta \left(k\right)+\lambda \left(k\right)\left[P_{D_{\tau (k)}}\left(\theta \left(k\right)\right)-\theta \left(k\right)\right]. \end{array}$$(3)Stopping rule: ∥θ(k+1)−θ(k)∥≤ϵ. where *ϵ* is a predefined threshold.


From the above theorem, we can see that the POCS algorithm has good convergence performance in the consistent case of CFP. However, the convergence behavior of POCS in the inconsistent case is generally unsatisfactory. A new relaxation sequence $\left\{\lambda {\left(k\right)}_{\left(k\geq 0\right)}\right\}$ needs to be used, which is given by:

(27) $$\begin{array}{c} \sum_{k=0}^{+\infty }\lambda \left(k\right)=+\infty ,\lambda \left(k+1\right)\leq \lambda \left(k\right),\lim_{k\to +\infty }\lambda \left(k\right)=0. \end{array}$$

By using the relaxation sequence stated above, the POCS will converge to a point in *G*, which has been verified by simulation in [38], but no theoretical analysis is provided.

Incremental subgradient optimization method For the sequential distributed algorithms, in [36], the authors proposed an incremental optimization algorithm called the normalized incremental subgradient algorithm. Since in their algorithm, a simple subgradient operator is adopted, hence it is hard to avoid the local optima solutions. The localization performance is low even though the authors claim and prove the convergence of the proposed algorithm.

One issue of the sequential distributed algorithm is that a specified data transmitting path is required. How to do path planning in a large sensor network is challenging. Furthermore, the convergence rate is low when the sensor density is high, and data transmission becomes unreliable when some of the nodes fail. Hence, the robustness of such networks is low.

#### 3.2.3. Fully-Distributed Algorithm

To avoid the path planning problem in the sequential distributed method and improve the robustness of the network, a fully-distributed method is useful, in which no fusion center is required and sensor nodes only need to communicate with their closest neighbors, therefore reduces the probability of congestion around the sink nodes and increases the robustness of the network against node failures or unpredictable switches to sleeping mode. Note that data transmission path planning is not needed for a distributed method.

Before introducing the proposed method, we give a brief introduction to a diffusion network. Let us represent the network as an undirected graph defined by G:=(N,E), where N is the node set N:=1,...,N and E⊆N×N is the edge set. If node *k* and node *l* can communicate with each other directly, we define the undirected link by (k,l)∈E.

If the CFP of the source localization problem is consistent, a fully-distributed protocol that can be used for source localization can be found in [55]. It works as follows: sensor *i* at time step k+1 generates its estimate according to the following protocol:

(28) $$\begin{array}{c} \theta^{i}\left(k+1\right)=P_{D_{i}}\left(\sum_{j=1}^{N}w_{j}^{i}\left(k\right)\theta^{j}\left(k\right)\right), \end{array}$$

where $w_{j}^{i}\left(k\right),i=1,...,N,j=1,...,N$ denote the weights at time step *k*; $\theta^{i}\left(0\right),i=1,...,N$ are arbitrary.

**Assumption** **1.** (Network connectivity) The network is connected, i.e., there exists a direct or indirect path between any two nodes.

**Assumption** **2.** *(Weighting rule) W(k) whose i-th row is the vector*

$$w_{i}\left(k\right)=\left[w_{1}^{i}\left(k\right),...,w_{N}^{i}\left(k\right)\right],$$

*is an N×N doubly-stochastic weighting matrix with the following properties:*

(29) $$\begin{array}{c} 1^{T}W\left(k\right)=1^{T},W\left(k\right)1=1. \end{array}$$

In real applications, actually, it is hard to know whether the CFP of the source localization problem is consistent or not. In this case, a new distributed localization protocol needs to be designed:

(30) $$\begin{array}{ccc} \theta^{i}\left(k+1\right) & = & \sum_{j=1}^{N}w_{j}^{i}\left(k\right)\theta^{j}\left(k\right)+\beta \left(k\right)\left[P_{D_{i}}\left(\sum_{j=1}^{N}w_{j}^{i}\left(k\right)\theta^{j}\left(k\right)\right)\right.\\ & & \left.-\sum_{j=1}^{N}w_{j}^{i}\left(k\right)\theta^{j}\left(k\right)\right], \end{array}$$

where β(k) is the relaxation sequence and $w_{j}^{i}\left(k\right)$ is the same as in Equation (28). We can see that if $\beta {\left(k\right)}_{k\geq 0}=1$, then the protocol Equation (30) is the same as Equation (28). The relaxation sequence β(k) plays an important role in the convergence of the method. Next, we study the case when β(k)≠1. The details will be given below.

**Assumption** **3.** $\sum_{k=1}^{\infty }\beta \left(k\right)\left(1-\beta \left(k\right)\right)=\infty ,\;\beta \left(k+1\right)\leq \beta \left(k\right),\;\lim_{k\to +\infty }\beta \left(k\right)=0.$

**Theorem** **2.** If Assumptions 2 to 4 hold, then by using the proposed fully-distributed protocol Equation (30), the location estimates of all sensors will converge to the optimal minimizer of Equation (23) [5].

**Figure 3:**
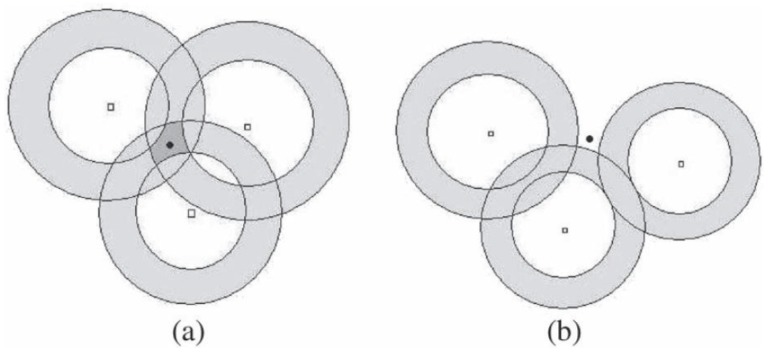
Intersection of rings. (**a**) consistent case; (**b**) inconsistent case [56].

Ring-based problem formulation: In [56], similar to the problem formulation shown in Figure 2, the authors formulate the localization problem as the intersection computation of a group of sensing rings as shown in Figure 3. In this work, the non-convex problem is converted into two weighted convex optimization problems.

Similarly, the authors also consider two cases: the consistent case and the inconsistent case. For the consistent case, the problem can be formulated as finding a point in the intersection set of the sensing rings. However, due to the existence of noises, there is still some possibility for the source to be located outside of the rings.

In [56], the authors propose to use a distributed protocol (refer to Equation (8) in [56]), which is the same as Equation (30). In this work, with alternating the computing of the projection on the inner and outer disks of each sensor, by using Protocol Equation (30), the estimates of all sensors converge to the optimal solution of the designed ring intersection optimization problem.

The projection-based method stated above is a good choice for real implementation in a centralized or distributed manner. However, it is hard to claim its accuracy theoretically, since it only considers bounded noise cases.

### 3.3. Summary for Single Source Localization

In the above, we have provided a detailed literature review for the energy-based single source localization problem. As we can see, plenty of algorithms have been proposed, including centralized, sequential and fully-distributed algorithms. In this subsection, we give a brief summary for the single source case. For the single source localization, ML-based algorithms, weighted least-squares, SDP, SOCP, subgradient, distributed projection, etc., can be used as a solution.

Generally speaking, centralized algorithms have a low robustness to network failures. In the designed algorithms, the fusion node is required to collect all measurements from others, and hence, communication congestion may occur during the data transmission. In the sequential algorithms, the sensor nodes compute the source location sequentially. Still, the network robustness is low. The fully-distributed algorithms have their advantage: robustness and easy implementation. For the localization accuracy performance, since in the projection-based distributed algorithms, only parts of Gaussian noise information are used for the derivation, it is hard to evaluate and compare the accuracy theoretically. However, simulation results in [5,56] have reported that the localization accuracy of projection-based methods is comparable to ML-based centralized algorithms.

#### 3.3.1. Localization Accuracy

**Figure 4:**
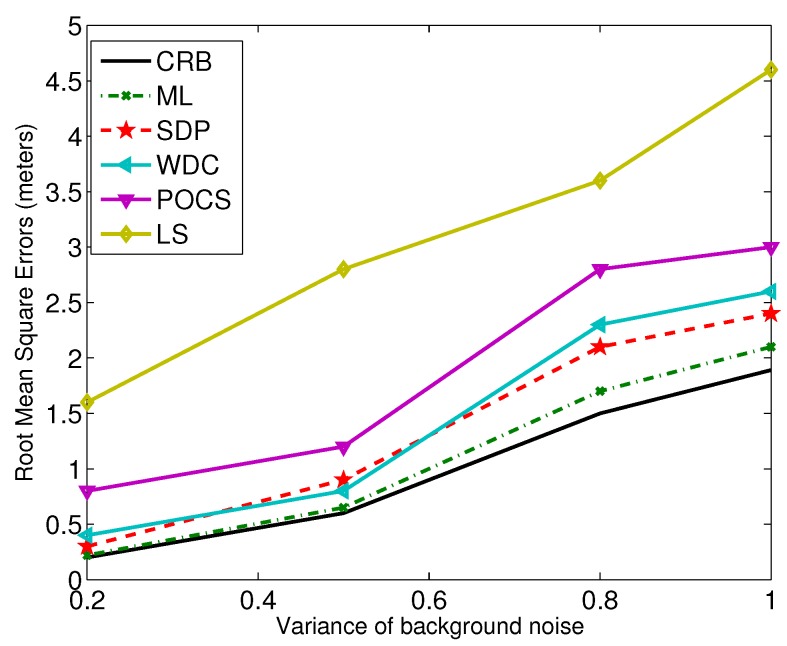
The noise levels versus RMSE. WDC, weighted direct least-squares method with correction; SDP, semidefinite programming; POCS, projection-onto-convex set.

**Figure 5:**
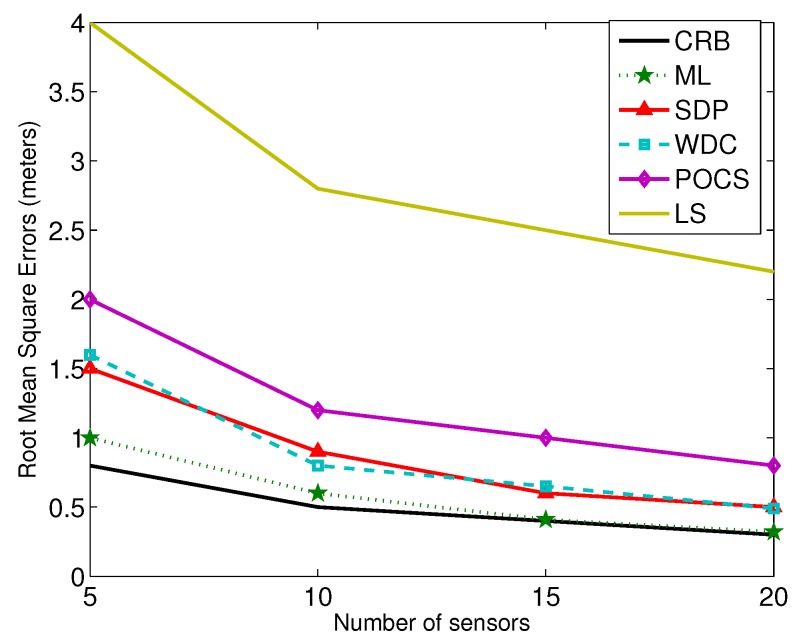
The number of sensors versus RMSE.

For the localization accuracy performance, generally, centralized algorithms, e.g., weighted least-squares, grid search, SDP, are better than POCS and its fully-distributed version [5,56]. Since in the projection-based distributed algorithms, only parts of Gaussian noise information are used for derivation, it is hard to evaluate and compare the accuracy theoretically. To compare all of the methods fairly, we give an example test environment. A number of *N* sensors are placed randomly and uniformly in the region of interest sized 50 m × 50 m. In the simulations, we vary the noise level and the number of the sensors in the field. The comparison results are shown in Figure 4 and Figure 5. In the figures, ML represents the grid search (with a 1×1 meter-sized grid), and LS represents the unconstrained least-squares method.

From the results, we can see that the SDP and WDC methods outperform the POCS and LS-based ones. For the POCS method, the noise term is ignored even though global optimization can be achieved. LS uses the general linear least-squares strategy, and the relationship between the unknown parameters is not considered.

#### 3.3.2. Computational Complexity

For the computational complexity, for the grid search, its complexity is $O(N^{3})$. For the WDC-based methods, it is reported that the complexity is at the level of $O(N^{2})$. SDP- or SOCP-based methods usually have a larger computational complexity. They both have a complexity in the order of $O(N^{3.5})$. For POCS-based methods, its computational complexity is the lowest, which is in the order of O(N). However, it will also depend on the number of iterations upon the convergence of the POCS.

#### 3.3.3. Communication Burden

For the centralized methods, all of the sensor measurements have to be transmitted to a fusion center or one of the sensors. For the fixed quantized level of energy estimates, $y_{i}$ and sensor coordinates, conveying the information from the sensors to a fusion center requires the transmission of O(N) bits over a distance of O(1) per bit [38]. However, for POCS-based ones, O(N) bits are transmitted over a distance of $O(\log^{2}L/L)$ per bit. Hence, it can be seen that the communication burden grows linearly with the number of sensors in the centralized approaches and sublinearly ($\sqrt{L}\log L$) in the decentralized approach.

Generally speaking, in sensor networks, it has been shown if the application and the sensor architecture permit, it is much more energy efficient to perform distributed local processing than to do central processing that requires extensive communication [4]. Of course, not all algorithms can use distributed/decentralized processing. It has been reported that POCS [38] and WDC [37] can be run in a distributed way.

#### 3.3.4. Implementation Issue

As we reported in this survey paper, most of the works done in the literature are algorithm developments. In these works, computer simulations are normally used to verify the corresponding algorithm performance in terms of complexity, accuracy, power consumption, etc. In view of the real implementation issue, the SDP algorithm is hard to implement in a large sensor network, since it can only be run in a centralized way. Distributed convex optimization methods, such as POCS, are easier to implement in a centralized, sequential or fully-distributed manner. In addition, POCS can be used for a large sensor network. LS-based methods, i.e., WDC, can also be implemented in a larger sensor network compared to SDP, but still, LS methods can be run in a fully-distributed manner. Hence, for the real implementation, POCS-related methods are highly recommended due to their high robustness to the network failures.

## 4. Multiple Source Localization: Algorithms and Analysis

In this section, we introduce the methods to solve the energy-based multiple source localization problems. The multiple source localization problem is more difficult since the sensor measurement is a superimposition of energy signals emitted from multiple sources. The number of unknown variables is quite large, and hence, an efficient number of sensors should be deployed to take the measurements.

We consider a signal model as in [7]. We assume that a total of *N* sensors is deployed, and *K* static acoustic sources are present in the sensor field, where *K* is the known number of acoustic sources. A fusion center is used to collect the measurement data of the sensors and to run the source localization algorithm. The locations of sensors, denoted by $r_{i}={\left[x_{i},y_{i}\right]}^{T},i=1,...,N$, are known to the fusion center. The locations of sources, denoted by $r_{sk}={\left[x_{sk},y_{sk}\right]}^{T},k=1,...,K,$ are unknown. The signal energy received by sensor *i* at time *t* is:

(31) $$y_{i}\left(t\right)=y_{si}\left(t\right)+w_{i}\left(t\right)=g_{i}\sum_{k=1}^{K}\frac{S_{k}\left(t\right)}{d_{ik}^{\alpha }\left(t\right)}+w_{i}\left(t\right),$$

where $d_{ik}=∥r_{i}-r_{sk}∥$ is the Euclidean distance between the *i*-th sensor and the *k*-th source; *α* denotes the unknown decay factor with typical value in the range from two to four; $S_{k}\left(t\right)$ is the signal energy at one meter away from the *k*-th source; $g_{i}$ is the gain factor of the *i*-th sensor; $w_{i}\left(t\right)$ is a Gaussian measurement noise with mean *μ* and variance $\sigma^{2}$, i.e., $w_{i}\left(t\right)∼N\left(\mu ,\sigma^{2}\right)$.

In the following, the time *t* is omitted for brevity. We define the following matrix notations.

$$\begin{array}{ccc} Y & = & {\left[y_{1}-\mu ,y_{2}-\mu ,...,y_{N}-\mu \right]}^{T},\\ H & = & diag\underset{N}{\underset{︸}{\left\{1/\sigma ,...,1/\sigma \right\}}},\\ D & = & \left[\begin{array}{cccc} \frac{g_{1}}{d_{11}^{\alpha }} & \frac{g_{1}}{d_{12}^{\alpha }} & \ldots & \frac{g_{1}}{d_{1K}^{\alpha }}\\ \frac{g_{2}}{d_{21}^{\alpha }} & \frac{g_{2}}{d_{22}^{\alpha }} & \ldots & \frac{g_{2}}{d_{2K}^{\alpha }}\\ \vdots & \vdots & \ddots & \vdots \\ \frac{g_{N}}{d_{N1}^{\alpha }} & \frac{g_{N}}{d_{N2}^{\alpha }} & \ldots & \frac{g_{N}}{d_{NK}^{\alpha }} \end{array}\right],\\ S & = & {\left[S_{1},S_{2},...,S_{K}\right]}^{T},\\ \varepsilon & = & {\left[\varepsilon_{1},\varepsilon_{2},...,\varepsilon_{N}\right]}^{T}, \end{array}$$

where $\varepsilon_{i}=\left(w_{i}-\mu \right)/\sigma ∼N\left(0,1\right),i=1,2,...,N$, are independent Gaussian random variables. *T* means the transpose of a matrix.

Using these matrices, Equation (31) can be rewritten as:

(32) HY=HDS+ε.

In addition, we denote:

(33) $$R=\mathrm{DS}={\left[\sum_{k=1}^{K}R_{1k},\sum_{k=1}^{K}R_{2k},...,\sum_{k=1}^{K}R_{Nk}\right]}^{T}\in R^{N\times 1},$$

where $R_{ik}=g_{i}\frac{S_{k}}{d_{ik}^{\alpha }},i=1,...,N.$

Then, the joint probability density function of Y can be expressed as:

(34) $$f\left(Y|\theta \right)={\left(2\pi \right)}^{-N/2}\exp\left(-\frac{1}{2}{\left(\mathrm{HY}-\mathrm{HR}\right)}^{T}\left(\mathrm{HY}-\mathrm{HR}\right)\right),$$

where:

$$\theta ={\left[r_{s1}^{T},r_{s2}^{T},...,r_{sK}^{T};S_{1},S_{2},...,S_{K};\alpha \right]}^{T},$$

and *α* is the decay factor.

The negative log-likelihood function is proportional to the quadratic form:

(35) $$\ell \left(Y|\theta \right)={\left(\mathrm{HY}-\mathrm{HR}\right)}^{T}\left(\mathrm{HY}-\mathrm{HR}\right),$$

which can be expressed as:

(36) $$\ell \left(Y|\theta \right)={\left(Y-R\right)}^{T}J^{-1}\left(Y-R\right),$$

where $J^{-1}=H^{T}H$ and $J=diag\underset{N}{\underset{︸}{\left\{\sigma^{2},...,\sigma^{2}\right\}}}.$

### 4.1. Multiresolution Search Method

A straightforward method to find a solution that maximizes the likelihood function is exhaustive search. However, the computation cost is extremely high, especially when there are multiple sources. For example, let there be *K* sources and *q* grid points to be searched in each dimension. Then the total number of search points with a two-dimensional sensor field will be equal to $q^{2K}$. While the computation complexity may be feasible for a desktop computer, it is likely to be excessive for low power sensor nodes with limited computing capabilities.

In [7], a multiresolution (MR) search method is proposed to mitigate the exponentially-growing computation complexity. Among several choices, a logarithmic MR search strategy will examine only *w* points in each dimension per iteration, where $q=w^{m}$, with *m* being the number of iteration. In each iteration, only $w^{2K}$ grid points needs to be searched. Then, another iteration of the search will be confined in the neighborhood of the current best solution by subdividing the coarser mesh around the current solution into *w* subdivisions and performing the search. After *m* iterations, the MR method will search at a grid size equal to that of the exhaustive search. However, the total search points will be $m\times w^{2K}$ rather than $q^{2K}=w^{2mK}$.

Obviously, due to the coarser search grid at the earlier iterations, the MR method may be trapped in a local minimum and yields an inferior solution.

### 4.2. Expectation-Maximization Algorithm

In order to further reduce the computational complexity and improve the localization performance, an efficient expectation-maximization (EM) algorithm for multiple source localization is proposed in [11].

Basic idea: For the EM algorithm, the basic idea is to decompose the aggregative energy signal into individual components and then estimate the corresponding location parameters separately for each source. The algorithm starts with the initialization based on a sequential dominant-source (SDS) scheme described in the next subsection. Each iteration consists of an E-step, an M-step and a decay factor parameter update step. In the E-step, we decompose the received energy of sensors to get the hidden data, which represent the signal energy received by the sensors from a source. In the M-step, a search method will be used to get the optimal source location estimate. In this work, an incremental parameterized search refinement scheme, i.e., the *β*-parameterized search method, is used. After getting the source location estimates, source energy can be computed accordingly. Then, using the estimated source energy information and location information, the decay factor can be obtained by using the normalized incremental gradient method. The details of the implementation of the algorithm can be found in [11].

### 4.3. Summary for Multiple Source Localization

According to our knowledge, there is not much work done in the energy-based multiple source localization problem as summarized in Table 1. The main reason is that the problem formulated is hard to solve, especially in an optimal way. The MR search-based method can be seen as a direct way to solve the problem. In this method, exhaustive search in a multiresolution manner is adopted to find solutions to minimize the log-likelihood function in Equation (36). Clearly, this kind of search method has a high computational complexity.

Instead, the improved EM algorithm proposed in [11] first decomposes the received energy from multiple sources by the sensors. Then, the incremental search method is applied for single source estimation. Since, in the search phase, the effort is only made for a single source, the computing resources are greatly saved.

## 5. Conclusions and Potential Research Directions

In this paper, a comprehensive review of energy-based single and multiple source localization algorithms was given. Firstly, we gave a brief introduction about the existing work on centralized, sequential and fully-distributed algorithms for the single source localization problem. Generally speaking, most of the existing algorithms are approximated ML estimation methods; for example, the SDP methods, POCS, WLS, etc. Simulation results were provided to compare these existing methods under different view points, such as computational complexity, communication issues, implementation issues, etc. For the multiple-source localization problem, the MR search method and EM algorithm are introduced briefly. Intuitively, the MR search method can work, but its computational complexity is high. The EM algorithm performs well if the initialization is good enough.

### 5.1. Discussion on Field Experiments

For field experiments for energy-based approaches, actually in the literature, not much work has been done. In [57], by using real corrupted or noisy signals collected from acoustic sources, such as a car, a helicopter and speech, source energy is then calculated. Simulation experiments are conducted by using ML-based methods.

Measurement noise and the real communication channel will be different from our assumption in this paper. To improve the localization accuracy in real field implementations, the noise model and energy decay factor need to be considered carefully. Model uncertainty, data uncertainty and environment uncertainty are three key issues to be incorporated in the problem formulation for real implementations. Perhaps the EM algorithm can be used to estimate the unknown parameters, especially for the energy decay factor [58].

Time synchronization and synchronization errors caused may be a big challenge compared to signal-based approaches; since for the energy-based approaches, the time duration for averaging has to be consistent among the sensors. Processing speeds for the sensor computing processors are different. This will also be challenging to localize and track sources with higher moving speeds.

### 5.2. Future Research Directions

For the future research directions, the following points are worth noting:

- Although much work has been done on the source localization of wireless sensor networks using static sensors, mobility is still one of the lesser explored aspects of this field. If sensors are mobile, few papers have dealt with the issue. A possible research direction is to share mobility information among sensors. Furthermore, if sensors can measure relative velocity to the source, this information can also be used to improve the localization accuracy.
- For the multiple-source localization problem, the researchers may consider combining two or more different types of sensor measurements. In addition, one interesting research direction is to simultaneously localize several different types of acoustic sources, i.e., several different animals, cars, etc.
- For energy-based acoustic source localization, it is interesting to mount the acoustic sensors to certain useful platforms to demonstrate the algorithms in real environments; for example, miniature quadrotors, warships, etc.

## Figures and Tables

**Table 1:** Multiple source localization problem. MR, multiresolution.

Algorithms	Representing Works	Localization Accuracy	Computational Complexity
MR search	[7]	Achieving CRB	Exponential with the number of sources
Improved EM algorithm	[11]	Achieving CRB	Linear with the number of sources
